# An investigation of microstructural, magnetic and microwave absorption properties of multi-walled carbon nanotubes/Ni_0.5_Zn_0.5_Fe_2_O_4_

**DOI:** 10.1038/s41598-019-52233-2

**Published:** 2019-10-29

**Authors:** Muhammad Syazwan Mustaffa, Raba’ah Syahidah Azis, Nor Hapishah Abdullah, Ismayadi Ismail, Idza Riati Ibrahim

**Affiliations:** 1 Department of Physics, Faculty of Science, Universiti Putra Malaysia, 43400 Serdang, Selangor Malaysia; 2 Functional Devices Laboratory, Universiti Putra Malaysia, 43400 Serdang, Selangor Malaysia; 3 Materials Synthesis and Characterization Laboratory, Universiti Putra Malaysia, 43400 Serdang, Selangor Malaysia

**Keywords:** Nanoscale materials, Soft materials

## Abstract

The enhancement of microwave absorbing properties in nickel zinc ferrite (Ni_0.5_Zn_0.5_Fe_2_O_4_) via multiwall carbon nanotubes (MWCNT) growth is studied in this research work. Ni_0.5_Zn_0.5_Fe_2_O_4_ was initially synthesized by mechanical alloying followed by sintering at 1200 °C and the microstructural, electromagnetic and microwave characteristics have been scrutinized thoroughly. The sintered powder was then used as a catalyst to grow MWCNT derived from chemical vapor deposition (CVD) method. The sample was mixed with epoxy resin and a hardener for preparation of composites. The composite of multi-walled carbon nanotubes/Ni_0.5_Zn_0.5_Fe_2_O_4_ shown a maximum reflection loss (*RL*) of −19.34 dB at the frequency and bandwidth of 8.46 GHz and 1.24 GHz for an absorber thickness of 3 mm for losses less than −10 dB. This acquired result indicates that multi-walled carbon nanotubes/Ni_0.5_Zn_0.5_Fe_2_O_4_ could be used as a microwave absorber application in X-band.

## Introduction

The high magnetic permeability, high resistivity and low eddy current loss of nickel zinc ferrite (Ni_0.5_Zn_0.5_Fe_2_O_4_) in the high-frequency region has made them an important candidate in soft magnetic material. One of the significant applications of this ferrite in the high frequency region is its high potential EM wave absorption properties. Ni_0.5_Zn_0.5_Fe_2_O_4_ exhibit good microwave absorbing performance due to its comparative properties to other ferrite^1,2^. Nevertheless, high density and poor temperature stability bound its application as a material for radar absorber in stealth aircraft and other ranges^3^. Recently, various efforts to develop microwave absorbing material in lower dimension have also been undertaken by many researchers to meet the requirements of microwave absorption applications by summarizing the structure and electronic state of 2D materials, and comprehensively overview their electromagnetic properties and response mechanisms^4–7^. As for carbon nanotubes (CNTs) which possess greater surface area and more dangling bonds causing an interfacial polarization and macroscopic quantum tunnel effect, have shown potential microwave absorbing performance^8^. Besides, the lightweight, good heat, corrosion and thermal shock resistance, higher thermal and electrical conductivity of CNTs advantage them as an auspicious candidate for advanced composite usage^9–11^. Previous research has shown that various percent of CNT introduction into soft and hard ferrite via sol-gel method, *in situ* precipitation, hydrothermal and *in situ* solvothermal has significantly improved the microwave absorption characteristics. The introduction of CNT into ferrite samples also has increased the conductivity^12–16^. In the present research work, multi-walled carbon nanotubes/Ni_0.5_Zn_0.5_Fe_2_O_4_ was synthesized using chemical vapour deposition (CVD) by using sintered Ni_0.5_Zn_0.5_Fe_2_O_4_ powder as a catalyst to investigate the impact of hybridization between magnetic and dielectric part towards the electromagnetic and microwave properties of the composite.

## Experimental Procedure

### Preparation of Ni_0.5_Zn_0.5_Fe_2_O_4_

All chemicals in this work were analytical grade reagents and used as raw materials without further purification. Nickel oxide, NiO (99.99%), zinc oxide, ZnO (99.99%) and iron oxide, Fe_2_O_3_ (99.95%) were provided and purchased from Alfa Aesar (Ward Hill, Massachusetts, U.S.). Ni_0.5_Zn_0.5_Fe_2_O_4_ has been prepared using Spex8000D milling apparatus for 2 h and the milled powders have been sintered for 10 h at a temperature of 1200 °C.

### Preparation of multi-walled carbon nanotubes/Ni_0.5_Zn_0.5_Fe_2_O_4_

**Figure 1:**
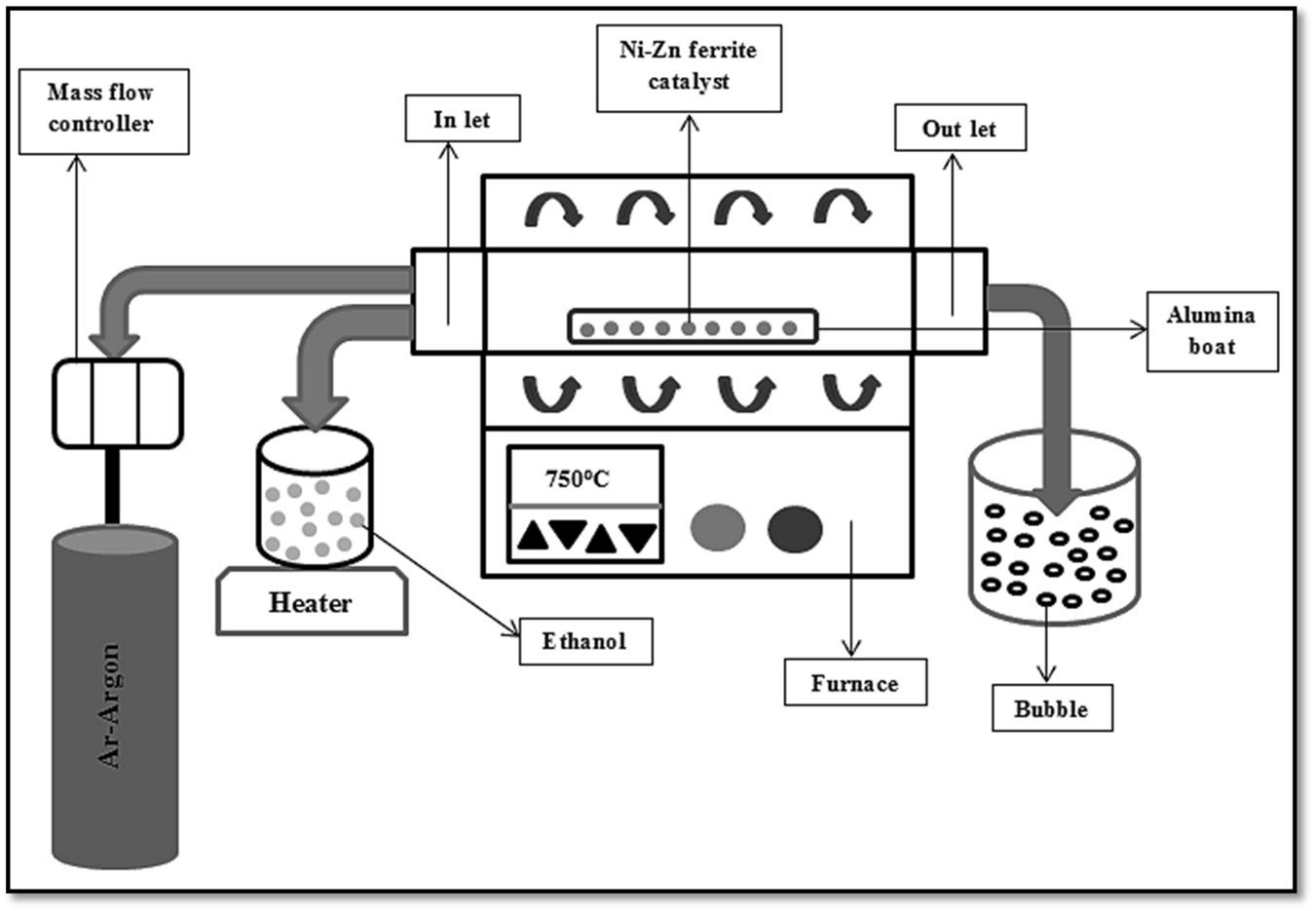
Schematic diagram of chemical vapor deposition (CVD).

Sintered powder of Ni_0.5_Zn_0.5_Fe_2_O_4_ was acting as a catalyst while an ethanol, C_2_H_5_OH (96%, Sigma Aldrich) solution as a carbon source in order to grow multiwall carbon nanotubes (MWCNT) via chemical vapor deposition (CVD) process (Fig. 1). The furnace was closed after placing an alumina boat that contained 0.6 g of Ni_0.5_Zn_0.5_Fe_2_O_4_ sintered powder with argon flushed at 100 sccm. Once the furnace reaches the targeted synthesis temperature at 750 °C, the evaporated ethanol solution at 100 °C temperature flowed for 30 min. After the process of flowing ethanol was completed, the furnace was left to cool down until room temperature before the sample was taken out for analysis.

### Preparation of multi-walled carbon nanotubes/Ni_0.5_Zn_0.5_Fe_2_O_4_ composite

The composite samples were produced by blending multi-walled carbon nanotubes/Ni_0.5_Zn_0.5_Fe_2_O_4_ powders with an epoxy resin (Araldite 506, Sigma Aldrich) in a 60:40 weight ratio. The mixture was poured in the different rectangular-shaped sample holder (model WR 90) of 2 and 3 mm thickness and being dried overnight in room temperature.

### Characterization

The phase identification was determined using X-ray diffraction, XRD (Philips X’pert Diffractometer model 7602 EA Almelo) with CuKα radiation (*λ* = 1.5406 Å). The surface morphology and elemental composition of the samples were observed by field emission scanning electron microscopy, FESEM (FEI Nova NanoSEM 230) fortified with an energy-dispersive X-ray, EDX (Oxford Instruments) system. Microwave and electromagnetic (EM) wave properties of the Ni_0.5_Zn_0.5_Fe_2_O_4_/MWCNT composites were performed via vector network analyzer, VNA (PNA N5227A) in the frequency range of 8 to 12 GHz.

## Results and Discussion

### Phase and microstructural analysis

**Figure 2:**
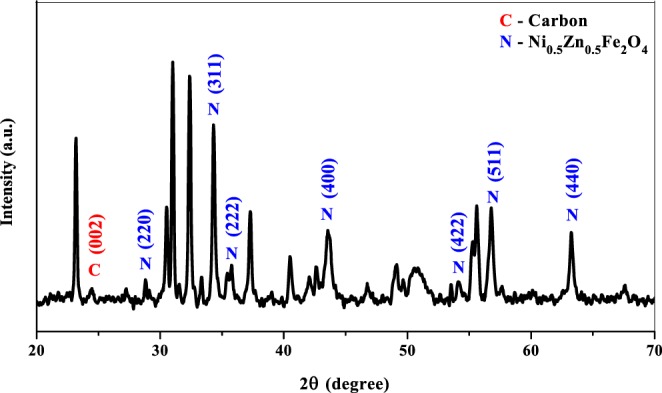
XRD pattern of multi-walled carbon nanotubes/Ni_0.5_Zn_0.5_Fe_2_O_4_.

Figure 2 showing the XRD pattern of multi-walled carbon nanotubes/Ni_0.5_Zn_0.5_Fe_2_O_4_. The appearance of the diffraction peaks at 2θ = 28.8°, 34.4°, 35.8°, 43.6°, 54.2°, 56.8° and 63.3° which corresponds to (220), (311), (222), (400), (422), (511) and (440) crystal planes, in consistent with the database found in the JCPDS file No. 08-0234, specify the formation of single-phase cubic of Ni_0.5_Zn_0.5_Fe_2_O_4_ with no extraordinary phase peaks. The observed diffraction peak appeared around 2θ = 24.6° is corresponds to the graphite (002) plane of MWCNT, which confirms the retaining of MWCNT structure without any destruction^17^. The existence of graphite lattice plane in XRD pattern indicates that the MWCNT were successfully synthesized by implementing Ni_0.5_Zn_0.5_Fe_2_O_4_ as a catalyst to grow the MWCNT.

**Figure 3:**
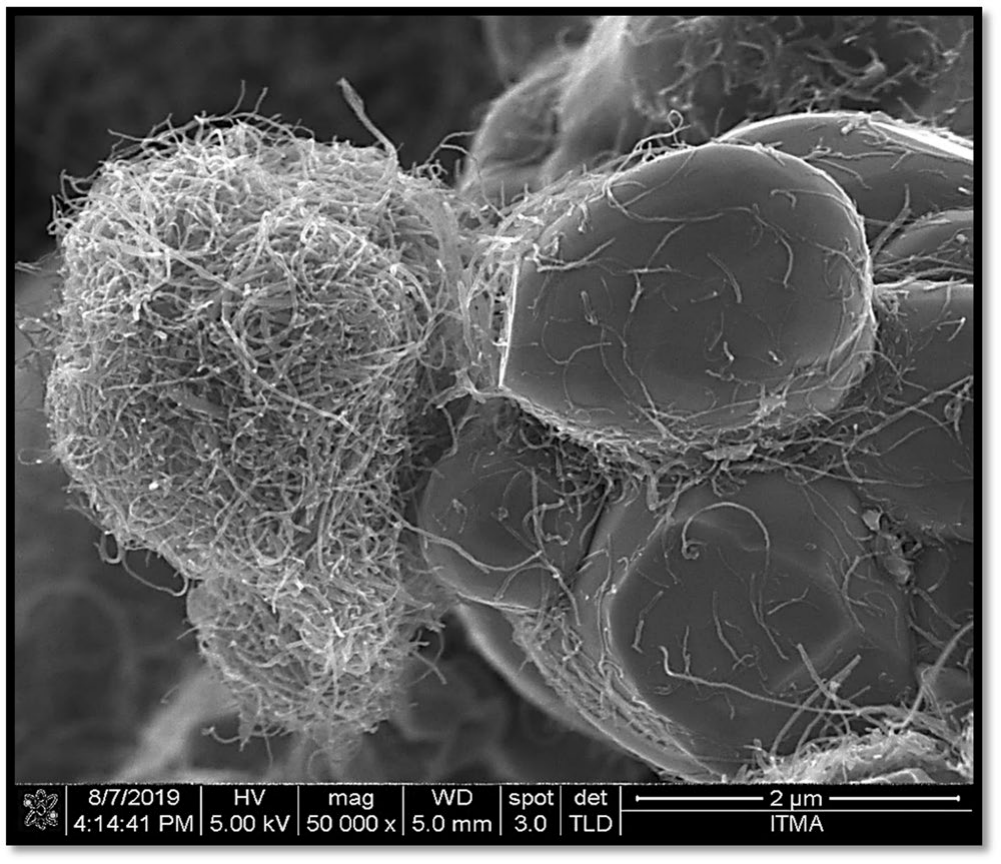
FESEM image of multi-walled carbon nanotubes/Ni_0.5_Zn_0.5_Fe_2_O_4_.

Figure 3 displays the FESEM image of multi-walled carbon nanotubes/Ni_0.5_Zn_0.5_Fe_2_O_4_. The outer diameter of MWCNTs is approximately ~50 nm. It should be noted that a huge amount of MWCNTs were deposited and dispersed almost co nsistently on the outer surface of nickel zinc ferrite particles. These surface functional groups could strongly bind to metal ions through an electrostatic attraction and assist as nucleation precursors.

**Figure 4:**
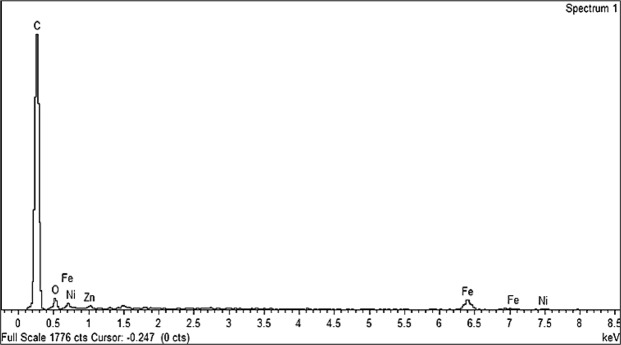
EDX pattern of multi-walled carbon nanotubes/Ni_0.5_Zn_0.5_Fe_2_O_4_.

Energy dispersive X-ray (EDX) was carried out in order to justify the chemical composition of prepared sample. The EDX spectra clearly revealed only existence of zinc (Zn), nickel (Ni), carbon (C), iron (Fe) and oxygen (O) peaks with no other contaminating constituent as can be perceived in Fig. 4.

### Electromagnetic (EM) analysis

**Figure 5:**
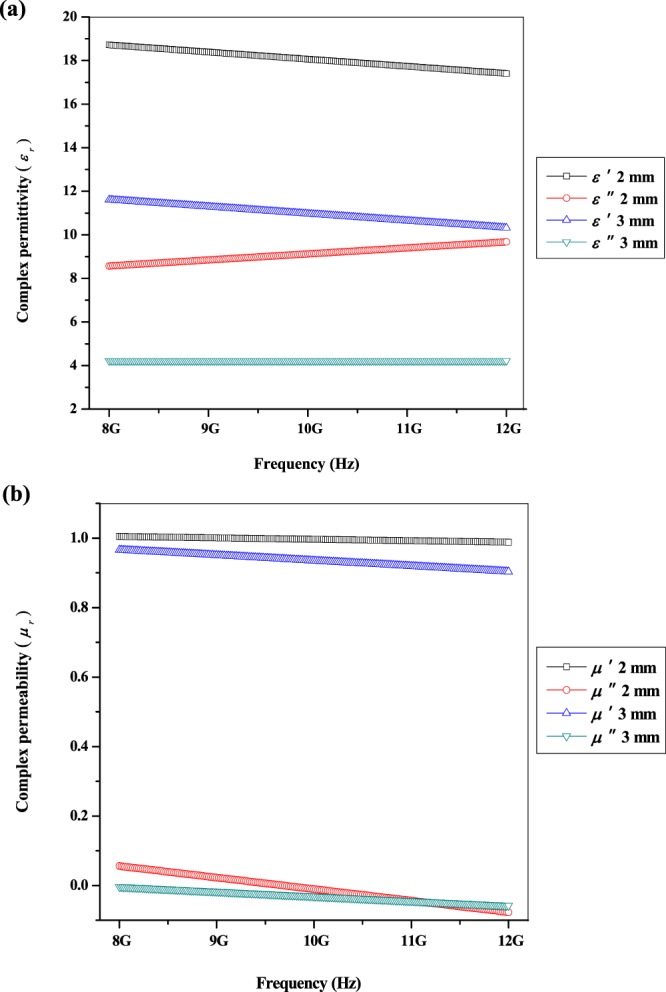
(**a**) Complex permittivity of multi-walled carbon nanotubes/Ni_0.5_Zn_0.5_Fe_2_O_4_.

Microwave absorbers are characterized by their electric permittivity and magnetic permeability as exposed in Fig. 5(a,b). The permittivity is a measure of the material’s effect on the electric field in the EM wave and the permeability is a measure of the material’s effect on the magnetic component of the wave. The permittivity is complex and is generally written as:

1 $${\varepsilon }_{r}=\varepsilon ^{\prime} -j\varepsilon ^{\prime\prime} $$

Analogous to the electric permittivity is the magnetic permeability which is written as:

2 $${\mu }_{r}=\mu ^{\prime} -j\mu ^{\prime\prime} $$

The imaginary parts (*μ*″, *ε*″) denote the dissipation of magnetic and electric energies while the real parts (*μ*′, *ε*′) represent the storage capability of magnetic and electric energies with both of them are frequency dependent. The material’s effect on the wave is entirely identified if the complex permeability and permittivity were known over a frequency range. Figure 5(a) illustrates the complex permittivity graph of multi-walled carbon nanotubes/Ni_0.5_Zn_0.5_Fe_2_O_4_ at different thicknesses. It can be observed that values of *ε*′ and *ε*″ were about 4 to 19 with no significant changes in the whole range of frequency (8–12 GHz). The *ε*′ values indicates a decreasing trend while the *ε*″ values show an increasing trend versus frequency changes which is due to the dipolar/orientation polarization mechanism^18–23^. When the EM wave from the network analyzer penetrates through the sample, it will manages to rotate and align the dipole moment with the field finds itself later colliding with another molecule and losing its alignment. At a higher thickness (3 mm), the EM energy to stimulate the dipole moment was decreased due to longer path for the EM to travel. Therefore, only slightly dipole is capable of being immobilized causing *ε*′ and *ε*″ values to be reduced relative to the sample that having lower thickness (2 mm)^21–23^. In addition, the *ε*′ and *ε*″ value at 2 mm thickness was higher than 3 mm thickness since it inversely proportional relation as confirmed by Eq. 3^24^:

3 $$\varepsilon ^{\prime} ={(1+\frac{\Delta \varphi {\lambda }_{0}}{360d})}^{2}\,{\rm{and}}\,\varepsilon ^{\prime\prime} =\frac{\Delta \varphi {\lambda }_{0}\sqrt{\varepsilon ^{\prime} }}{8.686\pi d}$$

where *ε*′ is real permittivity of sample, *ε*″ is imaginary permittivity of sample, *λ*_*o*_ is guided wavelength, *d* is sample thickness and ∆*ϕ* is phase difference between incident and reflected waves.

The complex permeability of multi-walled carbon nanotubes/Ni_0.5_Zn_0.5_Fe_2_O_4_ at different thicknesses is shown in Fig. 5(b). It can be found that *µ*′ and *µ*″ values were around 0.9 to 1.0 and −0.5 to 0.05 with a decreasing trend from low to high frequency range. The decreasing trend values of *µ*′ and *µ*″ were attributed to the relaxation of magnetization induced by domain wall displacement at lower frequency and spin rotation at upper frequency in the samples. At a lower thickness (2 mm), the EM energy to magnetize the magnetic (spin) moment was increased due to shorter route for the EM propagation. Thus, the *µ*′ and *µ*″ values at 2 mm thickness were enhanced compared to sample that having higher thickness (3 mm) and can be related with the Eq. 4^25^:

4 $$\delta =\frac{1}{\sqrt{\pi f{\mu }_{0}{\mu }_{r}\sigma }}$$

where *δ* is skin depth (thickness) of sample, *f* is the frequency, *µ*_0_ is the permeability of free space (4π × 10^−7^ H/m), *μ*_*r*_ is the relative permeability of sample and *σ* is the electrical conductivity.

**Figure 6:**
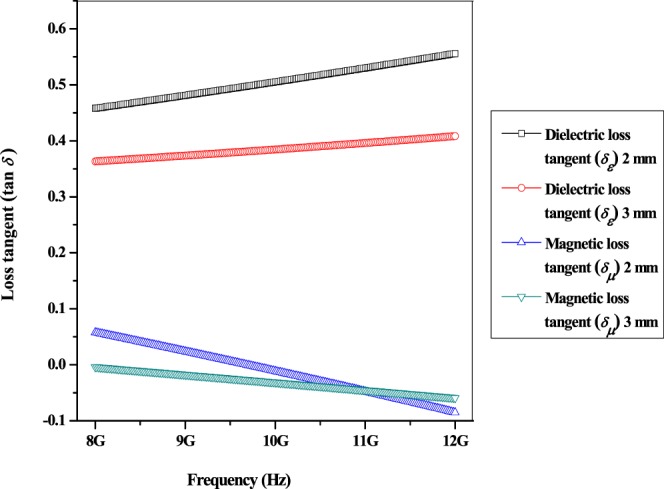
Dielectric and magnetic loss tangent of multi-walled carbon nanotubes/Ni_0.5_Zn_0.5_Fe_2_O_4_.

Figure 6 demonstrated the magnetic loss tangent (tan *δ*_*µ*_) and dielectric loss tangent (tan *δ*_*ε*_) of multi-walled carbon nanotubes/Ni_0.5_Zn_0.5_Fe_2_O_4_, respectively. The value of tan *δ*_*ε*_ lies within the range of 0.35 to 0.55 while tan *δ*_*µ*_ have an average value from −0.1 to 0.05. The tan *δ*_*ε*_ and tan *δ*_*µ*_ at 2 mm thickness have higher value than 3 mm thickness due to more energy had been attenuated with lower storing energy that infiltrate into the material as can be defined with Eq. 5 ^26^:

5 $$\tan \,{\delta }_{\varepsilon }=\frac{\varepsilon ^{\prime\prime} }{\varepsilon ^{\prime} }\,{\rm{and}}\,\tan \,{\delta }_{\mu }=\frac{\mu ^{\prime\prime} }{\mu ^{\prime} }$$

where *ε*′ is real permittivity, *ε*″ is imaginary permittivity, *µ*′ is real permeability and *µ*″ is imaginary permeability.

The multi-walled carbon nanotubes/Ni_0.5_Zn_0.5_Fe_2_O_4_ composites retain a higher tan *δ*_*ε*_ at 8–12 GHz which implies that the EM microwave absorption is contributed by dielectric loss mechanism rather than magnetic loss mechanism^18–20^.

### Microwave absorption analysis

**Figure 7:**
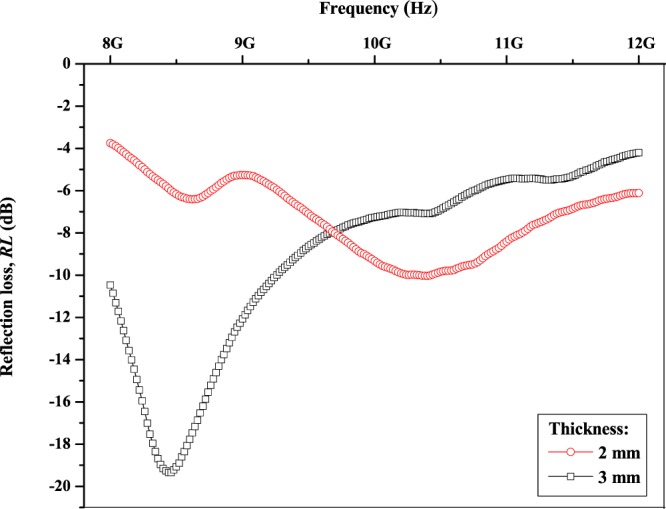
Reflection loss (*RL*) of multi-walled carbon nanotubes/Ni_0.5_Zn_0.5_Fe_2_O_4_.

Figure 7 shows the measured *RL* of the multi-walled carbon nanotubes/Ni_0.5_Zn_0.5_Fe_2_O_4_ composite with difference thicknesses of 2 and 3 mm respectively in the 8–12 GHz frequency range. The multi-walled carbon nanotubes/Ni_0.5_Zn_0.5_Fe_2_O_4_ gained the maximum absorption loss with *RL* −19.34 dB with a bandwidth of 1.24 GHz at a frequency of 8.46 GHz on the thickness of 3 mm which manifests optimum ability of microwave absorption (Table 1). The microwave absorption at 3 mm has been perceived to be increased compared to prior thickness of 2 mm as a result of better impedance matching between dielectric and magnetic loss of a material^19–23^. In addition, the absorption peak in this sample was attributed to the existence of both dielectric and magnetic resonance at the similar frequency. Generally, the EM wave absorbing characteristics were calculated using Eqs 6 and 7^20–23,27–29^:

6 $$R({\rm{dB}})=20\,\log |\frac{{Z}_{in}-1}{{Z}_{in}+1}|$$

which *Z*_*in*_ is specified by

7 $${{\rm{Z}}}_{{in}}=\sqrt{\frac{{\mu }_{r}}{{\varepsilon }_{r}}}\,\tanh \,[j\frac{2\pi }{c}\sqrt{{\mu }_{r}{\varepsilon }_{r}}ft]$$

where *Z*_*in*_ denotes an absorber input impedance, *c* is the speed of light, *t* is thickness of sample, *f* is microwave frequency, *ε*_*r*_ is relative complex permittivity and *µ*_*r*_ is relative complex permeability. The resonance frequency, *f*_*m*_ was discovered to deflect towards lower frequency as the thickness of the sample was increased due to indirectly proportional relation as agreed to the Eq. 8^19–23,29,30^:

8 $${f}_{m}=\frac{{\rm{c}}}{2\pi \mu ^{\prime\prime} t}$$

where *µ*″ is an imaginary part of permeability, *f*_*m*_ is resonance frequency with maximum *RL*, *c* is velocity of light and t is thickness of sample.

**Table 1:** EM wave absorption properties of multi-walled carbon nanotubes/Ni_0.5_Zn_0.5_Fe_2_O_4_.

Thickness of sample, *d* (mm)	Reflection loss, *RL* (dB)	Resonance frequency, *f*_*m*_ (GHz)	Bandwidth (GHz) (*RL* < –10 dB)
2	**10**.**03**	**10**.**40**	**0**.**08**
3	**19**.**34**	**8**.**46**	**1**.**24**

## Conclusion

Multi-walled carbon nanotubes/Ni_0.5_Zn_0.5_Fe_2_O_4_ was successfully fabricated by chemical vapour deposition (CVD) technique. The reflection loss peak exhibited the characteristics of a maximum loss of −19.34 dB at a frequency of 8.46 GHz, with a bandwidth of 1.24 GHz for losses less than −10 dB when the thickness of the sample was 3 mm due to the good compatibility of dielectric and magnetic properties in the sample. As a result, multi-walled carbon nanotubes/Ni_0.5_Zn_0.5_Fe_2_O_4_ samples have the characteristics as a suitable candidate for microwave absorber applications.
